# Dietary RISCO NUTRIFOUR multistrain multikingdom probiotic consortia improves meat quality carcass traits and growth efficiency in heat stressed broilers

**DOI:** 10.1038/s41598-025-17988-x

**Published:** 2025-10-03

**Authors:** Abdulaziz A. Al-abdullatif, Maged A. Al-Garadi, Mohammed M. Qaid, Abdulkareem M. Matar, Mohsen M. Alobre, Mohammed A. Al-Badwi, Gamaleldin M. Suliman, Elsayed O. Hussein

**Affiliations:** Department of Animal Production, College of Food and Agricultural Sciences, King Saud University, P.O. Box 2460, Riyadh, 11451 Saudi Arabia

**Keywords:** Breast meat quality, Broiler growth efficiency, Carcass traits, Feed efficiency, Heat stress, Multi-strain probiotic, Drug discovery, Microbiology, Zoology, Medical research

## Abstract

Heat stress (HS) compromises broiler productivity by depressing growth, meat quality, and physiological resilience. This trial evaluated the effects of a novel multi-strain probiotic solution, RISCO NUTRIFOUR (MSPS), in 288 Ross 308 broilers reared under standard environmental temperatures until day 28, followed by exposure to cyclic HS (35 ± 1^°^C, 09:00–17:00; 23 ± 1^°^C thereafter) until day 42. Birds were randomly allotted to six dietary treatments within the basal diet: MSPS at 1, 2, or 4 mL kg^-¹^; *Bacillus subtilis* (1 mL kg^-¹^); *S. cerevisiae* (1 mL kg^-¹^); or an unsupplemented control (*n* = 8 pens * 6 birds). At day 42, breast meat quality and carcass characteristics of heat-stressed broilers were assessed. Also, the performance index (PI), European production efficiency factor (EPEF), and feed efficiency (FE) of the production cycle were calculated. MSPS at 1 mL kg^-¹^ significantly (*p* < 0.05) enhanced PI (+ 18.5%), EPEF (+ 18.2%), and FE (+ 13.6%) *versus* the negative control. Also, cooking loss percentage fell by 40.5% and breast meat lightness (L^*^) and whiteness index increased (*p* < 0.05). MSPS improved ultimate pH and reduced core temperature 24 h post-mortem, indicating better metabolic recovery. No adverse effects on relative internal organ weights, texture, or sensory traits were noted (*p* > 0.05). GC-MS revealed that MSPS has antioxidant and antimicrobial metabolites that may underpin these responses. In conclusion, MSPS, particularly at 1 mL kg^-¹^, mitigates HS-induced detriments to meat quality and growth, resulting in improved PI, EPEF, and FE, offering a practical antibiotic-free strategy for the broiler industry in hot climates.

**Supplementary Information:**

The online version contains supplementary material available at 10.1038/s41598-025-17988-x.

## Introduction

 Broiler enterprises in hot-arid and tropical regions routinely face cyclic heat stress (HS), where diurnal house temperatures, above the thermo-neutral zone, spike to ≥ 35 °C for several hours^1,2^. Such conditions depress feed intake, compromise gut integrity, and accelerate post-mortem-ultimately downgrading meat quality and growth efficiency^3,4^. Projections for Saudi Arabia and neighboring climates indicate that such events will intensify, making nutritional countermeasures a research priority^5^.

For decades, in-feed antibiotics masked much of the HS-related productivity loss; however, global drug cross-resistance concerns now restrict their use^6,7^. Alternative growth promoters already in use include probiotics, prebiotics, synbiotics, and other natural additives^8^. Probiotic strain introduction into the gut in a practical and consumer-friendly way has emerged as a leading antibiotic-free option because it modulates the microbiota-gut-immune axis, achieves a healthy relationship between them, improves nutrient digestibility, and buffers oxidative stress^2,9^. They also inhibit pathogen colonization in the gut, such as *Clostridium perfringens* and Salmonella, via competitive exclusion^6,7^ and can improve growth rate and feed conversion^10^.

Yet single-strain probiotic products effects on carcass quality under HS remain inconsistent. Some studies report benefits for color, pH, and tenderness^6,11,12^ whereas others find no change^13^. This inconsistency is compounded by two factors: most trials evaluate single‑strain products (e.g., *Bacillus subtilis* “*B. subtilis”* or *Saccharomyces cerevisiae “S. cerevisiae”*), whose performance under HS is variable, and the majority assess growth or immune indices, with far fewer interrogating detailed meat quality endpoints, especially under realistic cyclic HS regimens. *B. subtilis* improves skeletal growth and health in some trials^14,15^. While *S. cerevisiae* fermentation products lower plasma corticosterone levels by 44.4% and heterophil/lymphocyte (H/L) ratios by 33.3% and improve feed conversion by 4.54%, they only marginally affect average bird weights of Cobb 500 male broilers^16^.

Recent evidence suggests that multi-strain, multi‑kingdom consortia outperform mono-strain probiotics through complementary enzyme production, wider ecological niches, and metabolite cross-feeding. A comparison of Bacillus (including *B. subtilis*, *B. coagulans*, *B. licheniformis*, and *B. indicus*) and Lactobacillus (*L. acidophilus*, *L. buchneri*, *L. plantarum*, and *L. rhamnosus*) cocktails with single-strains under chronic HS reported superior body weight gain, villus architecture, and antioxidant indices with the Bacillus and Lactobacillus probiotic mixed preparation^17^.

Likewise, broilers given a multi-strain probiotic (*Lactobacillus acidophilus*, *B. subtilis*, and *Clostridium butyricum*, with a concentration of 2 * 10^8^ viable spores/kg) under high animal protein concentrate conditions showed enhanced dressing percentages, overall performance, and livability; positively impacted gut histomorphology; promoted beneficial bacteria (Lactobacillus); and reduced low-density lipoprotein and harmful bacterial growth (*Escherichia coli*, Salmonella, and *Clostridium perfringens*) compared to birds receiving control^18^.

MSPS (RISCO–NUTRIFOUR), a novel probiotic, combines seven microorganisms drawn from three microbial kingdoms. Multi-strain probiotics have high levels of phenolic, flavonoid, and fatty acid compounds with demonstrated antioxidant and antimicrobial actions^19^. In probiotic trials, such metabolites could synergize with the live microbes to stabilize gut redox status and limit spoilage flora, outcomes rarely explored. Nevertheless, scarcely any study has combined bacteria, yeasts, and photosynthetic microbes in one preparation or benchmarked such a blend against single-strain comparators under HS. Thus, this study hypothesized that MSPS, owing to its multi-kingdom synergy and endogenous antioxidant metabolites, would enhance growth efficiency, improve breast meat quality and its physicochemical stability and sensory traits, and maintain carcass yields in broilers subjected to cyclic HS, with maximal benefits at dose levels of 1–4 mL kg^-1^ feed. Does dietary MSPS-at graded inclusion rates-attenuate HS-induced impairments in breast meat quality, carcass traits, and growth efficiency metrics of broilers more effectively than equivalent single-strain preparations of *B. subtilis* or *S. cerevisiae*?

Despite the theoretical advantages of using multi-kingdom consortia that include photosynthetic bacteria, no published study has benchmarked their effects against single-strain probiotics under a realistic cyclic HS regimen (35 °C × 8 h kg^-1^) while simultaneously quantifying carcass yield, detailed meat physico-chemistry, and growth efficiency metrics. Moreover, the metabolic fingerprints that might underpin these responses (e.g., phenolic esters) have not been linked to in vivo outcomes. This study aims to compare three MSPS inclusion levels (1, 2, 4 mL kg^-1^) with single-strains of *B. subtilis* and *S. cerevisiae* (1 mL kg^-1^) and an un-supplemented control in Ross 308 broilers under HS in to quantifying carcass characteristics, physiochemistry kinetics, texture profile, and sensory attributes, as well as computing performance index (PI), feed efficiency (FE), and European production efficiency factor (EPEF). In addition, relate GC-MS identified metabolites in MSPS to observed physiological outcomes. By integrating multi-strain microbiology, metabolite chemistry, and comprehensive meat quality analytics under a stringent HS model, this work offers a novel, antibiotic-free avenue to sustain broiler productivity in hot climates and fills a critical gap in probiotic research.

## Materials and methods

### Ethical approval

The use of animals and all experimental procedures were reviewed and approved by the Scientific Research Ethics Committee at King Saud University, in compliance with the national guidelines for animal care and welfare in Saudi Arabia (Approval code: KSU-SE-21-47).

### Housing birds and experimental design

A total of 288 one-day-old male Ross 308 chicks were obtained from Al Khumasia Company for the Feed and Animal Products hatchery, Riyadh, Saudi Arabia. Chicks were individually weighed and randomly assigned to one of six dietary treatments in 8 replicates of 6 broilers per replicate at the following rates:

T1-T3: Birds received 1, 2, or 4 mL MSPS/kg basal diet, respectively.

T4: Birds received 1 mL/kg *Bacillus subtilis* PB6.

T5: Birds received 1 mL/kg *Saccharomyces cerevisiae*.

T6: Birds received a basal diet with un-supplemented probiotic.

The MSPS product used in this study is a multi-kingdom and multi-strain consortium probiotic mixture commercially known as RISCO–NUTRIFOUR^®^ solution. MSPS is a contain *Bacillus substiles* (1 × 10^^9^ CFU/ml), *Saccharomyces cerevisiae* yeast (1 × 10^^5^ CFU/ml), Lactobacillus species harbinensis and Parabunchneri (1 × 10^^9^ CFU/ml each), Rhodopseudomonas species palustris and shaeroides (1 × 10^^7^ CFU/ml each), and *Candida ethanolic* (1 × 10^^5^ CFU/ml). Colony-forming units (CFU)/ml and functional roles and key attributes of each microbial species of probiotic MSPS are described in Supplementary Table S2. MSPS is produced by Al Raya Specialties Industrial Factory in Riyadh, Saudi Arabia. The *Bacillus subtilis* PB6 is commercially available (CloSTAT^®^ 1:128, Kemin Industries, Inc., Des Moines, IA) and contains 1 × 10^9^ CFU/ml *Bacillus subtilis* spores according to the manufacturer^20^. *Saccharomyces cerevisiae* contains 1 × 10⁵ CFU/ml *(*SafMannan, Phileo, Lesaffre, Marcq-en-Baroeul Cedex, France). T4 and T5 served as positive controls, while T6 was used as the negative control.

Birds were fed a basal diet at three stages: a starter (1–14 d), a grower (15–28 d), and a finisher (29–42 d). The dietary rations were in mash form and formulated as per Aviagen^21^ as described in Supplementary Table S1.

Chicks were reared under standard environmental conditions until day 28. Upon arrival, the brooding temperature was set at 33 °C and gradually decreased by approximately 0.5 °C per day to reach 23 ± 1 °C by day 22, which was then maintained through day 28. During this period, relative humidity was maintained between 50 and 60%. From day 29 to 42, birds were subjected to a cyclic heat stress regimen (35 ± 1 °C from 09:00 to 17:00, followed by 23 ± 1 °C thereafter).

This study represents a distinct experimental phase following our previous 28-day trial^22^ conducted under the same ethical approval in line with institutional policy on multi-phase studies. While the earlier work addressed early-life responses under thermoneutral conditions, this study uniquely evaluates broilers exposed to cyclic heat stress from days 29 to 42, focusing on meat quality, carcass traits, and key performance indices.

Each cage measured 58 cm in length, 50 cm in width, and 35 cm in height, providing a total floor area of 0.29 m². Broilers were housed at a density of six birds per cage, equating to approximately 0.048 m² of floor space per bird. The birds were fed and watered *ad libitum* during the trial. All chicks were raised in environmentally controlled battery cages at KSU’s Experimental Poultry Research Unit, following conventional management techniques. The incandescent lighting scheme ran continuously for 24 h during the first week and for 23 h Light: 1-hour Dark (23 L:1D) during the rest of the weeks of the trial.

### Feed proximate analysis and MSPS bioactive chemicals

The nutrient composition of the feed samples was analyzed according to AOAC International methods^23^ used for crude protein (Method 990.03), crude fat (Method 920.39), fiber (Method 978.10), and ash (Method 942.05). Bioactive chemical composition mixtures of MSPS were separated using gas chromatography-mass spectrometry (GC-MS) as described in our previous study^22^. Al-abdullatif, et al.^22^ found that GC-MS profiling of MSPS extract revealed high levels of phenolics such as phenol (9.2%) and 2,4-di-tert-butylphenol (2,4 DTBP; 6.9%) and flavonoids such as trimethyl 1,2,3-propanetricarboxylate (9.52%) and some lipid (approximately 9.4%) fraction rich in palmitic (C16:0), stearic (C18:0), and omega-6 linoleic acid fatty acids with demonstrated antioxidant, anti-quorum sensing (anti-QS), and antimicrobial actions.

### Sample collection

On day 42, one broiler was randomly selected from each pen (totaling 8 birds per treatment) for sample collection. Broilers were slaughtered following the traditional Islamic Halal Method as described by Shahdan, et al.^24^ which involved severing the jugular veins, allowing the birds to bleed for 2 min, followed by semi-scalding at 54 °C for 30 s before manual plucking. Evisceration was carried out manually, and carcass components were subsequently estimated. Following draining, the *pectoralis major* was excised from each broiler. Then, samples were stored under two conditions: at 3 °C ± 0.5 °C for 1 and 24 h for physicochemical parameters (pH, core temperature, and color) and at −20 °C for meat quality measurements by following the traditional farm-fresh meat procedure.

### Meat quality assessments

#### pH and core temperature

The core temperature of the pectoralis major was recorded at 15 min and 24 h postmortem with a calibrated thermocouple thermometer. At the same time points, the pH was measured at three different positions within each fillet using a microprocessor pH meter (model pH 211, Hanna Instruments, Woonsocket, RI, USA) that had been calibrated with pH 4 and pH 7 buffers^25^. The average of the three readings was taken as the sample pH.

#### Color (CIE L∗ a∗ b∗)

Fillet color was measured on the internal surface at two different locations, 15 min and 24 h after slaughter, with a CR-400 Chroma Meter (Konica Minolta, Tokyo, Japan) following the CIE^26^ L∗ (lightness), a∗ (redness), and b∗ (yellowness) system^25^. Briefly, three random readings per site were averaged to obtain lightness (L*), redness (a*), and yellowness (b*) in accordance with American Meat Science Association (AMSA) guidelines^27^. From these values, chroma, hue angle, delta color change (∆E), browning index (BI), and whiteness index (WI) were calculated as described by Cázares-Gallegos, et al.^28^ to give a fuller perception of consumer-relevant color attributes.

#### Water-holding capacity (WHC)

WHC% was estimated based on the meat weight loss when a pressure is applied to the muscle by the compression method^25^. Briefly, approximately 2–3 g of muscle cut from a standardized location was sandwiched between pre‑weighed filter papers and acrylic plates and compressed under a 10 kg load for 5 min. Weight loss was calculated in duplicate, and WHC was expressed as the percentage of exuded water relative to the initial sample weight^29^.

#### Cooking loss (CL)

CL% was assessed in an air‑forced oven preheated to 170 °C as described in Pelicano, et al.^25^. The internal temperature of the meat inside the oven was logged using a data logger (Thomas Scientific), and samples were removed at 75 °C. CL (%) was calculated as the difference between raw and cooked weights, as described by Hussein, et al.^30^.

#### MFI

MFI, an indirect indicator measure of calpain proteolysis activity according to Suliman, et al.^31^. Four grams of thawed, minced muscle were homogenized in 40 mL of ice‑cold MFI buffer (2 °C) for 30 s using a blender, washed repeatedly with MFI buffer, and the absorbance of a 0.5 mg/mL suspension was read at 540 nm using a spectrophotometer (HACH DR/3000, Loveland, CO, USA). MFI = Absorbance (A_540_) of the resulting 0.5 mg/mL solution * 200.

#### Texture: shear force (SF) and texture profile analysis (TPA)

Tenderness was measured with a TA‑HD Texture Analyzer (Stable Micro Systems Ltd., Godalming, UK). Five rectangular cores (2 * 1 * 1 cm) cut parallel to the muscle fibers from each cooked sample were sheared with a Warner-Bratzler blade; peak force (N) was recorded as SF. For TPA, the analyzer, fitted with a cylindrical probe, compressed the same samples to 80% of their original height in two cycles, yielding hardness, cohesiveness, chewiness, and springiness values^32^.

#### Sensory evaluation

Breast meat samples were roasted at 200 °C to an internal temperature of 75 °C, coded, and served warm to a trained panel following Pelicano, et al.^25^. Fifteen trained male panelists (aged 30–45 years) participated in the sensory evaluation. All panel members had a minimum of one year’s experience in sensory evaluation of food and demonstrated competent performance. Each participant received individually coded meat samples, which were served sequentially. Prior to the evaluation, the sensory attributes and scoring procedures were clearly explained to the panelists, following the method described in^33^. Sensory evaluation was conducted across five independent test sessions, during which a total of 48 meat samples were assessed for sensory attributes as descriped in^34^. Using a nine‑point hedonic scale, panelists scored flavor, texture, preference, and general appearance; mean scores for each attribute were reported.

#### Growth, feed, and economic efficiencies

Performance index (PI), feed efficiency (FE), and the European production efficiency factor (EPEF) are key indicators used to evaluate broiler production, each emphasizing a different aspect of performance and economic efficiency. PI = (Final body weight/Age)/Feed conversion ratio “FCR”. FCR = Feed consumption/gain. A higher PI reflects both better growth and efficient feed utilization^35^. FE is a measure of how birds convert feed into weight gain more efficiently. FE = Total weight gain/Total feed intake. A higher FE is desirable. EPEF is a key performance indicator calculated in the broiler industry to assess the economic efficiency of growth^36^. EPEF = (Viability (%) * Body weight (kg) * 100)/(Age (days) * FCR). A higher EPEF and a lower FCR are desirable.

### Statistical analysis

Statistical analyses were performed in accordance with the ARRIVE 2.0 guidelines to ensure transparent and reproducible reporting of animal research. The experimental design followed a completely randomized structure, with dietary treatment as the sole fixed factor. The experimental unit was the pen for performance index and feed efficiency metrics, and a randomly selected bird per pen (*n* = 8 per treatment) was used for carcass and meat quality assessments. Sample size was determined based on previous studies^37–39^ with 8 replicates per treatment ensuring sufficient replication and maintaining statistical power and logistical feasibility to detect biologically meaningful differences (α = 0.05). To enhance the robustness of data and improve statistical power, multiple technical replicates were performed per biological unit. For physicochemical parameters (e.g., pH, temperature, and color), three readings were taken from each meat sample and averaged. For water-holding capacity (WHC), two technical replicates were performed per sample. Sensory evaluation was conducted across five replicate sessions, with each of the 48 samples assessed by trained panelists for juiciness, tenderness, flavor, and overall acceptability. These repeated measurements helped reduce measurement error and increase the precision of treatment comparisons, thereby strengthening the reliability of the statistical inferences drawn. Allocation to treatment groups was randomized using a computer-generated random sequence. All investigators and laboratory personnel involved in outcome assessment were blinded to treatment groups during data collection and analysis. Outcome measures were assessed for normality (Kolmogorov–Smirnov test) and homogeneity of variance (Levene’s test). When assumptions were met, data were analyzed using one-way ANOVA via the General Linear Model (GLM) procedure in SAS 9.4 (SAS Institute Inc., Cary, NC, USA). The statistical model used was:

$$\:{\gamma\:}_{ij}={\upmu\:}+{T}_{i}\:{+e}_{ij}$$

Where *Y*_*ij*_ is the observation, µ the overall mean, *T*_*i*_ is the fixed effect of the *i*^th^ treatment, and *e*_ij_ the residual error. When a significant main effect was detected (*P* < 0.05), means were separated with Tukey’s Studentized Range (HSD) test. Results are expressed as mean ± SEM; corresponding effect sizes (η²) and 95% confidence intervals are reported to facilitate interpretation. No missing-data imputation was required, and all statistical decisions were made a priori.

## Results

The nutrient content analysis of the basal diet is shown in Supplementary Table S3. Basal diets were rich in macronutrients, particularly nitrogen-free extract and crude protein.

### Carcass characteristics

As shown in Table 1, the dietary inclusion of MSPS in heat-stressed broilers did not significantly affect live weight, hot or cold carcass weights, or carcass yield (*p* > 0.05). Among the relative weights of carcass parts and internal organs, no significant differences (*p* > 0.05) were observed across treatments, although a trend toward lower gizzard weight was noted in the 1 mL/kg MSPS-fed group compared to controls (*p* = 0.087).

**Table 1:** Carcass traits of 42-day-old heat-stressed broilers fed a multi-strain, multi-functional RISCO-NUTRIFOUR (MSPS) probiotic compared to those fed single-strain probiotics.

Treatment	MSPS Levels (ml/kg)			1 ml/kg Bacillus subtilis	1 ml/kg Saccharomyces cerevisiae	Negative control	Standard error	*P* value
Item	1	2	4					
Live weight (kg)	2.72	2.93	2.84	2.69	2.85	2.92	0.075	0.138
Hot Carcass weight (kg)	1.82	1.92	1.89	1.81	1.89	1.96	0.053	0.294
Cold carcass weight (kg)	1.77	1.86	1.83	1.76	1.84	1.9	0.052	0.374
Carcass yield (%)	66.9	65.7	66.6	67.2	66.6	67.2	0.85	0.833
Relative weight (g/100 g hot carcass)								
Breast	40.4	39.7	40.8	39.6	39.4	46.9	2.38	0.226
Leg	40.2	40.7	39.5	39.3	40.3	40.3	0.653	0.655
Wings	7.33	7.41	7.2	7.4	7.69	6.91	0.235	0.319
Back bone	9.5	9.02	8.78	9.67	9.16	8.6	0.393	0.373
Liver	2.08	2.35	2.28	2.24	2.26	2.36	0.087	0.226
Proventriculus	0.476	0.429	0.471	0.426	0.422	0.422	0.027	0.516
Pancreas	0.298	0.295	0.271	0.242	0.284	0.274	0.016	0.16
Heart	0.703	0.757	0.663	0.673	0.675	0.659	0.029	0.174
Kidney	0.646	0.661	0.624	0.643	0.61	0.705	0.036	0.533
Gizzard	2.82	2.76	2.95	2.8	2.58	2.45	0.127	0.087

### Physicochemical properties of breast meat

The physiochemical characteristics of breast meat, such as pH, core temperatures, color components, and color derivatives of 42-day-old heat-stressed broilers fed basal diets with probiotic supplements, are presented in Table 2. Significant treatment effects were detected for both initial and ultimate breast meat pH values. MSPS supplementation at 2 and 4 mL/kg led to higher pH values at 1 h postmortem compared to the negative control and single-strain groups (*p* = 0.034). Also, MSPS supplementation at 2 mL/kg and *B. subtilis* at 1 mL/kg led to higher pH values at 24 h postmortem compared to the negative control group (*p* = 0.002).

**Table 2:** Physicochemical properties of breast meat measured 24 h postmortem of 42-day-old heat-stressed broilers fed a multi-strain, multi-functional RISCO-NUTRIFOUR (MSPS) probiotic.

Treatment	MSPS Level (ml/kg)			1 ml/kg Bacillus subtilis	1 ml/kg Saccharomyces cerevisiae	Negative control	Standard error	*P* value
Item	1	2	4					
Meat pH								
Initial pH	5.74^b^	5.93^a^	5.92^a^	5.77^b^	5.84^ab^	5.84^ab^	0.412	0.034
Ultimate pH	5.86^ab^	5.92^a^	5.89^ab^	5.91^a^	5.85^ab^	5.82^b^	0.684	0.002
Core temperature								
Initial temperature	29.5^ab^	28.6^b^	30.2^ab^	28.3^b^	31.0^a^	31.6^a^	0.05	0.001
Ultimate temperature	14.3^b^	14.1^b^	14.0^b^	15.4^a^	15.2^a^	15.5^a^	0.022	< 0.001
Ultimate color and its derivatives								
L*	49.2^a^	43.2^b^	46.4^ab^	45.0^ab^	43.0^b^	48.4^a^	0.048	0.001
a*	2.6	3.9	3.6	4.49	4.73	3.77	1.37	0.175
b*	16.3	14.6	14.4	16.1	16.4	15.4	0.607	0.087
∆E	46.7^b^	52.2^a^	49.2^ab^	50.8^ab^	53.0^a^	47.4^b^	0.645	< 0.001
Chroma	16.5	15.1	15.2	16.7	17.1	15.9	1.31	0.102
Hue	80.9	75.3	75.3	74.4	74	76.4	0.631	0.311
BI	43.4^b^	47.3^b^	42.4^b^	50.7^ab^	56.1^a^	43.6^b^	2.3	0.001
WI	46.6^a^	41.2^b^	44.2^ab^	42.5^ab^	40.4^b^	45.9^a^	2.75	< 0.001
a*/b* Ratio	0.16	0.265	0.278	0.281	0.287	0.243	1.29	0.344

*n* = 8 birds per group. ^a and b^ Values with different superscripts in the same row are significantly different (*p* < 0.05). Abbreviations: L*: Lightness; a*: Redness; b*: Yellowness. ∆E: Total ultimate color change; Chroma: Saturation index; Hue: Hue angle; BI: Browning index; WI: Whiteness index; a*/b* Ratio: Redness: Yellowness Ratio. .

Core temperature at 1 h postmortem showed significantly lower values in 2 mL/kg MSPS-treated birds and 1 mL/kg *B. subtilis-*treated birds compared to other groups (*p* = 0.001). In addition, core temperature at 24 h postmortem showed significantly lower values in MSPS-treated birds compared to the negative control and single-strain groups (*p* < 0.001).

Meat color lightness (L*), delta color change (∆E), BI, and WI were significantly influenced by MSPS supplementation (*p* < 0.001), with improved L* and WI values particularly in the 1 mL/kg MSPS group. No significant differences were observed in redness (a*), yellowness (b*), chroma, hue angle, or a*/b* ratio (*p* > 0.05).

### Meat quality parameters

Meat quality traits (WHC%, CL%, MFI, and SF) for breast samples of heat-stressed broilers measured at day 42 are presented in Table 3. CL% was significantly reduced in MSPS-supplemented birds, particularly at 1 mL/kg, compared to control and single-strain probiotic groups (*p* = 0.001), indicating better water retention and the most favorable values for CL%. No significant effects were observed for WHC%, MFI, or SF among treatments (*p* > 0.05). The MSPS-supplemented groups had suitable numerical SF (N) and MFI values, which showed the highest MFI and the lowest SF values compared to other groups.

**Table 3:** Meat quality parameters of 42-day-old heat-stressed broilers fed a multi-strain, multi-functional RISCO-NUTRIFOUR (MSPS) probiotic compared to those fed single-strain probiotics.

Treatment	MSPS Level (ml/kg)			1 ml/kg Bacillus subtilis	1 ml/kg Saccharomyces cerevisiae	Negative control	Standard error	*P* value
Item	1	2	4					
WHC (%)	73.7	72.6	73.5	72.6	72.6	71.5	1.06	0.458
CL (%)	16.6^b^	18.9^b^	17.1^b^	27.9^a^	27.7^a^	27.9^a^	1.13	0.001
MFI	109	108	105	106	106	107	3.29	0.956
SF (N)	3.46	3.57	3.44	3.89	3.83	3.89	0.157	0.116

*n* = 8 birds per group. ^a and b^ Values with different superscripts in the same row are significantly different (*p* < 0.05). Abbreviations: WHC: Water holding capacity, CL: Cooking loss, MFI: myofibril fragmentation index, and SF: shear force. .

### Texture profile analysis (TPA)

Texture attributes such as hardness, springiness, chewiness, and cohesiveness were not (*p* > 0.05) influenced by MSPS treatment (Table 4). Although higher springiness values with MSPS approached statistical significance (*p* = 0.05), this trend suggests that MSPS, particularly at 1 mL/kg, may enhance meat elasticity and resilience-qualities often linked to more desirable texture attributes such as reduced chewiness (requiring less force and fewer chews to break down the meat) and a more pleasant mouthfeel.

**Table 4:** Texture profile analysis of 42-day-old heat-stressed broilers fed a multi-strain, multi-functional RISCO-NUTRIFOUR (MSPS) and single-strain probiotics.

Treatment	MSPS Level (ml/kg)			1 ml/kg Bacillus subtilis	1 ml/kg Saccharomyces cerevisiae	Negative control	Standard error	*P* value
Item	1	2	4					
Hardness (N)	5.84	6.48	6.37	6.75	7.39	7.07	0.555	0.45
Springiness	0.899	0.858	0.824	0.8	0.808	0.794	0.044	0.05
Cohesiveness	0.547	0.535	0.64	0.603	0.617	0.627	0.681	0.552
Chewiness	2.82	2.86	3.43	3.28	4.28	3.72	0.03	0.101

*N* = 8 birds per group. .

### Sensory evaluation (SE)

Sensory attributes of broiler breast meat from heat-stressed birds subjected to different dietary treatments are shown in Table 5. SE scores for juiciness, tenderness, flavor, and overall acceptability were statistically similar across all treatments (*p* > 0.05). Nevertheless, broilers supplemented with MSPS at 1 mL/kg consistently received numerically higher scores across all sensory attributes- juiciness (7.00), tenderness (6.90), flavor (7.00), and general acceptability (7.20)-compared to other treatments. These trends suggest a potential enhancement in palatability and consumer appeal with MSPS supplementation under cyclic HS, particularly at the 1 mL/kg level.

**Table 5:** Sensory evaluation score of 42-day-old heat-stressed broilers fed a multi-strain, multi-functional RISCO-NUTRIFOUR (MSPS) and single-strain probiotics.

Treatment	MSPS Levels (ml/kg)			1 ml/kg Bacillus subtilis	1 ml/kg Saccharomyces cerevisiae	Negative control	Standard error	*P* value
Item	1	2	4					
Juiciness	7	6.17	6.17	6.27	5.75	6.13	0.365	0.296
Tenderness	6.9	6.33	6.08	6.17	6	6.13	0.357	0.521
Flavor	7	6.9	6.5	6.33	6.63	6.75	0.228	0.327
General acceptability	7.2	7.03	7.05	7.17	6.42	6.5	0.257	0.135

n = 8 birds per group.

### Growth and feed efficiencies

The efficiencies of growth and feed of heat-stressed broilers throughout the trial period are shown in Table 6. Broilers fed MSPS, particularly at 1 mL/kg, exhibited significantly higher PI (*p* = 0.012) and EPEF (*p* = 0.013), and the best FCR (*p* = 0.05) compared to the negative control and single-strain groups. Feed efficiency (FE) was also improved in MSPS-fed birds, particularly at 1 mL/kg (*p* = 0.049).

**Table 6:** Feed conversion ratio (FCR), performance index (PI), feed efficiency (FE), and European production efficiency (EPEF) factor of 42-day-old heat-stressed broilers fed a multi-strain, multi-functional RISCO-NUTRIFOUR (MSPS) probiotic compared to those fed single-strain probiotics.

Treatment	MSPS Levels (ml/kg)			1 ml/kg Bacillus subtilis	1 ml/kg Saccharomyces cerevisiae	Negative control	Standard error	*P* value
Item	1	2	4					
FCR	1.42^b^	1.44^b^	1.45^b^	1.49^ab^	1.48^ab^	1.51^a^	0.032	0.05
PI	52.6^a^	48.9^ab^	49.8^ab^	44.4^b^	45.6^b^	44.4^b^	1.78	0.012
FE	0.750^a^	0.700^ab^	0.720^ab^	0.670^b^	0.680^b^	0.660^b^	0.021	0.049
EPEF	533^a^	496^ab^	505^ab^	454^b^	463^b^	451^b^	18	0.013

^a−c^ Values with different superscripts in the same row are significantly different (*p* < 0.05). .

## Discussion

HS is widely recognized as one of the major ecological challenges in poultry production, as it significantly affects broiler growth, FE, and meat quality, thereby reducing overall production efficiency. HS at 35 ± 1 °C for 8 h day^-1^ clearly depressed growth and meat quality in the negative control broilers, confirming the severity of the challenge and echoing the multifaceted physiological burdens described in recent HS research^40–42^. Supplementation with the MSPS-comprising *B. subtilis*, *S. cerevisiae*, two *Lactobacillus* spp., two photosynthetic *Rhodopseudomonas* spp., and *Candida ethanolic* mitigated most of these detriments. The following sections place those outcomes in the context of prior single and multistrain probiotic research.

MSPS at 1 mL kg^-1^ elevated PI, FE, and EPEF by around 18.5, 13.6, and 18.2%, respectively, and improved FCR by 9-point versus negative control. Broilers with higher PI, FE, and EPEF indexes typically exhibit superior traits, such as faster growth and better feed conversion, which reduce costs and increase revenue. Each performance index point contributes approximately $0.20 in economic value. Comparable gains have been reported for individual MSPS constituents, such as Wang, et al.^43^ who found that Bacillus**-**based products improved body weight gain (BWG) and FCR in broilers exposed to 32 °C for 10 h day⁻¹ and able to cope with HS more effectively by attenuating HSinduced behavioral and inflammatory markers through regulation of microbiota-modulated immunity. Also, Sumanu, et al.^44^ found yeast derivatives (*Saccharomyces cerevisiae*) as anti-stress agents enhancing thermoregulation, lowering HS biomarkers (cloaca and surface temperatures), and enhancing growth and health under chronic HS, possibly leading to better FE and reduced dependence on antibiotics. Dietary antimicrobial peptide and probiotic supplements to broilers resulted in 23.93% and 19.70% higher BWG and 76.59% and 70.27% lower mortality, respectively, compared to the positive control^45^.

Furthermore, Xu, et al.^46^ observed that administration of *Rhodopseudomonas palustris* (8 × 10⁹ cells d^-1^) in drinking water improved daily BWG, FCR, lower fat content, meat quality, and sensory attributes of broilers during the whole growing period of 6 weeks. Yet most singlestrain trials report more modest or variable responses, suggesting that the synergistic consortium in MSPS is important. Several studies showed that multistrain formulations outperform monostrain products for growth and immune indices through enhanced biological activities, owing to complementary enzymatic profiles and broader colonization niches^47,48^. This study supports this notion that only the complete MSPS formulation-not its individual components, Bacillus or Saccharomyces-enhances growth efficiency and meat quality more effectively than single-strain alternatives. Although single-strain probiotics are better for growth and health, multi-strain probiotics may be even more beneficial due to synergistic and additive effects between the individual isolates^48^.

Neither MSPS nor the singlestrain probiotics altered carcass yield or organ weights, mirroring findings with *B. subtilis*^49^ and multistrain blends (*Bacillus coagulans* plus *Bacillus subtilis* plus *Saccharomyces boulardii*) supplied at 10^⁷^ CFU/g feed^47^. Imam, et al.^50^ found that banana tuber flour augmented with *S. cerevisiae* as β-glucan fiber in feed increased live weight and BWG but did not have a significant effect on carcass percentage and abdominal fat. Probiotic treatments decreased fat content^13^ and improved carcass yield^45^. Stability in relative organ mass implies that growth improvements stemmed mainly from enhanced nutrient utilization-likely via gutbarrier reinforcement and enzyme secretion-rather than from disproportionate tissue accretion.

Few studies have evaluated probiotic cocktails under HS while also measuring detailed meatquality endpoints. An earlier *Lactobacillus*based blend (32 °C, 10 h) increased ultimate pH but failed to affect CL or color^51^; MSPS therefore sets a new benchmark by delivering simultaneous gains in growth efficiency and technologically relevant meat attributes. The inclusion of photosynthetic bacteria and yeasts-both absent from most commercial blends-may be critical for this broader efficacy spectrum. Thus, MSPS sharply reduced CL by 40% and raised ultimate pH and breastmeat lightness/whiteness. These benefits exceed those seen with lacticacidbacteria mixtures, which sometimes fail to influence water holding or color under similar cyclic HS models^51^. In contrast with Suryadi, et al.^13^ who found probiotic treatments had no significant effect on meat pH. In partial agreement with Muneeb, et al.^45^ who found that probiotic supplements to the *necrotic enteritis*-affected broilers improved WHC, CL, dripping loss, and SF. The higher efficacy may arise from spore-forming *B. subtilis*, whose thermostable proteases accelerate postmortem proteolysis, elevating ultimate pH and water retention^52,53^. Also, yeast βglucans modulate systemic redox status, curbing HSinduced lipid oxidation and pigment degradation^16^. Furthermore, photosynthetic *Rhodopseudomonas* spp. are known to supply carotenoids and coenzyme Q, which enhance cellular antioxidant capacity and may stabilize sarcoplasmic proteins during chilling^54,55^.

Lipid oxidation diminishes meat flavor, color, and nutritional value; thus, phenolic and flavonoid antioxidants are routinely applied to poultry products^56^. In this trial, broilers given 1 mL/kg MSPS recorded the highest-but not statistically different-scores for juiciness, tenderness, flavor, and overall acceptability, mirroring earlier probiotic studies that reported subtle sensory gains under HS^57–59^. In line with Tan, et al.^60^, the group’s lower CL suggests improved water retention, a key driver of succulence and flavor release. GC-MS profiling helps explain these trends: MSPS contained potent antioxidants-phenol (9.2%) and 2,4‑di‑tert‑butylphenol (6.9%)-plus some lipid fraction (around 9.4%) rich in palmitic and stearic acids and ω‑6 linoleic acid. Phenolic quench reactive oxygen species, limiting drip and preserving color^61^, while saturated and ω‑6 fatty acids serve as flavor precursors during cooking^62^. Additional aroma metabolites from *S. cerevisiae* and *Candida ethanolic* may further enhance palatability. Overall, the numerical sensory advantage of the 1 mL kg^-1^ MSPS diet, together with its favorable physicochemical profile and antioxidant‑rich composition, indicates a practical potential to improve meat quality in HS broilers-even where statistical significance is not achieved.

The distinct microbial members of MSPS can act at multiple biological levels. At the gut-centric level, lactobacilli lower intestinal pH, reinforce tight junctions, and outcompete pathogens; *B. subtilis* secretes enzymes that liberate nutrients; and yeasts provide mannanoligosaccharides that bind enteric toxins. At the systemic level, microbial-derived phenolics (e.g., 2,4-DTBP), flavonoids (e.g., trimethyl 1,2,3-propanetricarboxylate), and carotenoids, which are produced by photosynthetic bacteria, deter oxidative damage, aligning with the diminished core carcass temperature and higher ultimate pH observed in this study. Immunomodulatory level, photosynthetic bacteria, and yeast βglucans skew cytokine profiles toward antiinflammatory pathways, which is pivotal under HSinduced immunosuppression^43,44^. While MSPS proved effective over a relatively short HS period (29–42 d), longer growouts and fieldscale trials are needed to confirm economic returns. Parallel gutmicrobiota and metabolomics analyses would clarify strainspecific contributions. Finally, dose–response work around the 1 mL kg^-1^ optimum could refine costbenefit ratios. Overall, the multistrain MSPS outperformed its singlestrain counterparts and many previously published probiotics in alleviating the twin challenges of HSinduced growth depression and meatquality deterioration. Its composite microbial ecology and rich pool of antioxidant metabolites appear to confer a multifaceted protective effect that warrants further commercial exploration in hotclimate broiler systems. MSPS-especially at 1 mL kg^-1^ improved growth efficiency and meat water retention during cooking while maintaining, and numerically enhancing, sensory acceptability under HS.

## Conclusion

In conclusion, exposure to daily cyclic HS between 29 and 42 d markedly reduced growth efficiency and compromised meat quality in untreated birds. Dietary inclusion of MSPS, especially at 1 mL kg^-1^, counteracted these effects by enhancing growth efficiency and production efficiency, meat quality, and maintaining carcass integrity. Dietary MSPS, especially at 1 mL kg^-1^ elevated PI, EPEF, and FE signify better growth under thermal stress, as well as lower CL, whiteness index and lightness breast meat, favorable ultimate pH, and core temperature, reflecting superior waterholding capacity and muscle metabolism. No adverse effects were observed on carcass yield or organ indices, with texture preserved and sensory acceptability numerically improved under HS. The bioactive composition of MSPS, rich in phenolics, flavonoids, and fatty acids, likely contributes antioxidant and antimicrobial actions that stabilize gut function and systemic homeostasis during HS. MSPS therefore represents a viable, antibiotic-free intervention to sustain broiler productivity and product quality in hotclimate production systems.

## Supplementary Information

Below is the link to the electronic supplementary material.

Supplementary Material 1

## Data Availability

The data that support the findings of this study are available on request from the corresponding author upon reasonable request.
