# Supplementary material for: Dietary RISCO NUTRIFOUR multistrain multikingdom probiotic consortia improves meat quality carcass traits and growth efficiency in heat stressed broilers
Source: Sci Rep. 2025 Oct 3;15:34566. doi: 10.1038/s41598-025-17988-x (PMC12494778; doi:10.1038/s41598-025-17988-x)
Supplement: Supplementary file 1 — Supplementary Material 1 [file 41598_2025_17988_MOESM1_ESM.docx]

# **Supplementary Tables**

**Supplementary Table S1.** Components of a base diet (%), separated by growth phase

| **Ingredients** | **Starter**  **(1-14 d)** | **Grower**  **(15-28 d)** | **Finisher**  **(29-42 d)** |
| --- | --- | --- | --- |
| Corn grain | 53.92 | 55.51 | 60.70 |
| Soybean meal 48 | 38.23 | 35.90 | 30.57 |
| Soybean oil | 3.44 | 4.65 | 5.08 |
| Limestone | 1.54 | 1.39 | 1.28 |
| Common salt | 0.38 | 0.38 | 0.38 |
| Vitamin premix | 0.10 | 0.10 | 0.10 |
| Mineral premix | 0.10 | 0.10 | 0.10 |
| DL-Methionine | 0.35 | 0.31 | 0.28 |
| L-Lysin HCL | 0.21 | 0.15 | 0.15 |
| L-Threonine | 0.14 | 0.10 | 0.08 |
| Mono calcium phosphate | 1.59 | 1.41 | 1.28 |
| Choline CL-60% | 0.003 | **-** | **-** |
| **Total** | **100.00** | **100.00** | **100.00** |
| **Calculated nutrient (%)** |  |  |  |
| Metabolic energy (Mega cal/kg) | 3.0 | 3.1 | 3.2 |
| Crude protein | 23.0 | 21.15 | 20.0 |
| Non phytate phosphor | 0.48 | 0.44 | 0.405 |
| Calcium | 0.96 | 0.87 | 0.81 |
| D- Lysine | 1.28 | 1.15 | 1.06 |
| Sulfur amino acids (methionine and cysteine) | 0.95 | 0.87 | 0.83 |
| Threonine | 0.86 | 0.77 | 0.71 |

**Supplementary Table S2*.*** *In vitro* multi-kingdom and multi-strain consortium present in probiotic RISCO–NUTRIFOUR solution and count of its colony forming units per ml (CFU mL⁻¹)

| **Kingdom** | **Species (CFU mL⁻¹)** | **Functional roles and key attributes of each microbial species** |
| --- | --- | --- |
| **Bacteria** | *Bacillus subtilis* (1 × 10⁹) | Thermostable enzymes, spore‑forming gut colonizer ^52,53^ |
|  | *Lactobacillus parabuchneri* (1 × 10⁹) | Lactic acid, tight‑junction protection ^63^ |
|  | *Lactobacillus harbinensis* (1 × 10⁹) | Antioxidant peptides, gut pH modulation ^64^ |
|  | *Rhodopseudomonas shaeroides* (1 × 10⁷) | carotenoids, coenzyme Q, N‑fixation ^65^ |
|  | *Rhodopseudomonas palustris* (1 × 10⁷) | Photo‑heterotrophy, vitamin synthesis; enhances growth and meat flavor in broilers ^46^. |
| **Yeasts** | *Saccharomyces cerevisiae* (1 × 10⁵) | β‑glucans, MOS, stress‑hormone suppression ^16^ |
| **Fungi** | *Candida ethanolic* (1 × 10⁵) | Produce ethanol and carbon dioxide as end‑products during ethanolic fermentation, synthesize B‑vitamins ^66^ |

**Supplementary Table S3*.*** Proximate composition (%) of macronutrients in the broilers' basal grower and finisher diets, expressed on a dry matter basis^1^

| Item | | Starter | Grower | Finisher |
| --- | --- | --- | --- | --- |
| Moisture | | 6.30 | 7.10 | 7.90 |
| Dry matter (DM) | | 93.70 | 92.90 | 92.10 |
|  | **% DM** | | | |
| Crude ash | | 6.43 | 5.71 | 4.99 |
| Crude protein | | 22.9 | 20.67 | 18.24 |
| Crude fat (EE) | | 8.43 | 8.61 | 8.79 |
| Crude fiber | | 3.42 | 3.12 | 2.82 |
| Nitrogen-free extract (NFE) | | 58.82 | 61.89 | 65.16 |
| **Total** | | **100** | **100** | **100** |

^1^ The analysis was performed in duplicate.
